# The association of locomotive and non-locomotive physical activity measured by an accelerometer with functional fitness in healthy elderly men: a pilot study

**DOI:** 10.20463/jenb.2018.0007

**Published:** 2018-03-31

**Authors:** Hyejoon Park, Wonil Park, Moran Lee, Nayoung Ko, Eunkyung Kim, Kazuko Ishikawa-takata, Jonghoon Park

**Affiliations:** 1. Department of Physical Education, Korea University, Seoul Republic of Korea; 2. Department of Food and Nutrition, Gangneung-Wonju National University, Gangneung Republic of Korea; 3. Department of Nutritional Education, National Institute of Health and Nutrition Republic of Korea

**Keywords:** elderly men, accelerometer, daily physical activity, functional fitness

## Abstract

**[Purpose]:**

The purpose of this study was to investigate the relationship of various aspects of daily physical activity, such as the number of steps, time spent in moderate to vigorous intensity physical activity (MVPA), and locomotive and non-locomotive MVPA measured by a triaxial accelerometer, with the functional fitness in healthy elderly men.

**[Methods]:**

The subjects of this study were 22 healthy elderly men aged over 65 years. The participants wore a triaxial accelerometer for two weeks to estimate their daily physical activities. The level of functional fitness was measured based on "National Fitness Award 100 in Korea" immediately after the measurement of two weeks of daily physical activities.

**[Results]:**

The results showed that active healthy elderly men with more than 6,500 walking steps per day and more than 60 min per day spent in MVPA showed a significantly higher 2-min marching in place and index of cardiorespiratory endurance compared to less physically active participants. Particularly, locomotive MVPA was significantly associated with cardiorespiratory endurance levels (r = 0.50), whereas non-locomotive MVPA was not associated with other measurements of functional fitness.

**[Conclusion]:**

Increased MVPA time, especially the locomotive MVPA, can effectively suppress the decrease in cardiorespiratory endurance level in elderly men. However, no association was observed between non-locomotive MVPA, such as household activities, and functional fitness in healthy elderly men.

## INTRODUCTION

According to the World Population Prospects 2017 by the World Health Organization, the elderly population is expected to increase globally, with elderly individuals over the age of 65 comprising 8.2% of the global population in 2015 to 17.6% in 2060^1^. Korea has become an aged society in 2000, when the proportion of the elderly population rose beyond 7% of the country’s population and has shown faster growth rates compared to other developed nations; going forward, this proportion of elderly individual above the age of 65 is expected to increase from 13.1% in 2015 to 40.1% in 2060 ^2^. Moreover, this increase in the number of elderly population will inevitably increase healthcare expense^2^. In an aged society, the social trend is to prefer higher quality of life and a healthy lifestyle rather than the length of lifestyle itself^3, 4^, with the question of "how can my post-retirement life be maintained?" being the most significant social issue. Multiple policies have been proposed on geriatric health for healthy post-retirement lifestyle^5, 6^; however, there is still a lack of research on physical activity, which is critical for the maintenance of good health for the elderly population.

With the advancement of science and technology making the human life more convenient and affluent, the lack of physical activity in elderly population and the resulting decrease in physical well-being have been reported as two of the most significant factors influencing elderly health^7^. Nam (2012) studied post-menopausal women using the International Physical Activity Questionnaires (IPAQ) to identify differences in the variables related to physical activity in daily life and health-related fitness. The results indicated that high physical activity group had better general endurance, muscular endurance, and flexibility compared to the low physical activity group^8^. Park et al. (2016) studied elderly women using the IPAQ questionnaires to review the relationship between the variables related to physical activity in daily life and daily living fitness. They reported that elderly individuals engaging in physical activity composed of 150 min of moderate physical activity or 75 min of vigorous physical activity had significantly higher lower- body muscular endurance and coordination^9^. However, the above studies used a survey and not an objective measurement tool for measuring physical activity; as such, they had the advantage of assessment of a large group of population in a small amount of time, but had the disadvantage of significantly decreased accuracy^10^. While multiple methods were proposed for measuring physical activity, the accelerometer with a speed sensor can objectively and accurately measure the number of steps taken, as well as the intensity of physical activity ^11, 12^.

Corcoran et al. (2016) used a triaxial accelerometer to identify the correlation between physical activity and fitness in elderly individuals aged over 65 residing in long-term care facility^13^. In this study, active elderly individuals had significant differences in cardiovascular and respiratory endurance and grip strength compared to their non-active counterparts. Yasunaga et al. (2017) studied elderly Japanese people aged over 65 years using a triaxial accelerometer to measure the time spent on moderate to vigorous physical activity (MVPA) and reported that MVPA time has a positive correlation with walking speed and balance; moreover, they reported that increasing MVPA by 10 min a day could increase fitness by 1.4–2.7% overall^14^. Lee et al. (2010), which studied Korean elderly people, reported that more time spent on light and moderate physical activity, exclusive of vigorous physical activity, led to a positive correlation with walking fitness, which was advantageous for preventing injuries from falls^15^. Moreover, Santos et al. (2012) reported that the daily time spent on MVPA for elderly individuals aged over 65 years, measured using a triaxial accelerometer, had a significant positive correlation with elderly fitness; additionally, they reported that a sedentary behavior such as sitting had a negative correlation with elderly fitness, independent of time spent on MVPA^16^. With advancing age, the walked distance decreases, leading to sedentary physical activity focused on sitting, which in turn it leads to an increase in non-locomotive physical activity such as those using the upper body^17^. In a previous study, we have developed a special algorithm to differentiate between locomotive and non-locomotive activity, in telling apart the volume and intensity of physical activity by dividing the upper and lower body. Normally, accelerometers are very effective in identifying the movements of the lower body that engage in walking; however, they underestimate physical activity performed by the upper body ^19^. Moreover, the data obtained from accelerometers were found related with lower-body strength and walking ability^14, 15^; however, some studies indicated a decreasing correlation between lower body strength to grip strength in elderly individuals compared to younger people. Therefore, it would be difficult to identify this relationship using a normal accelerometer that is specific in measuring physical activity such as walking. Therefore, the hypothesis of this study was that an accelerometer that differentiates the locomotive physical activity centering around the daily lives of elderly individuals from non-locomotive physical activity that centers around the upper body would be useful for identifying not only the cardiovascular endurance and walking ability, but also upper-body strength. To the best of our knowledge, no studies that have differentiated physical activity into locomotive and non-locomotive in order to identify correlations with various fitness-related factors in the elderly population are currently available.

The purpose of this study was to measure the variables of physical activity, such as the number of steps taken per day, MVPA, locomotive and non-locomotive physical activity in healthy elderly men and identify the relationships of these variables to elderly health.

## METHODS

### Subjects

The subjects of this study were elderly men aged > 65 years residing in downtown Gangneung, Gangwon Province; the subjects participating in this study did not engage in regular exercise, did not have any orthopedic conditions, and did not take drugs for treatment purposes. Prior to the experiment, we have explained the contents and the purpose of this study, obtained approvals to participate in the experiment, and engaged in measurements of physical activity relating to daily life and of fitness over two weeks during the end of April. We used 5-m walking test, an identifier of the body’s aging status, to identify 30 physically healthy elderly individuals. The data of 22 individuals were used in this study after excluding those who did not measure physical activity associated with their daily lives (*n* = 5) and those who did not measure their cardiovascular and respiratory endurance (*n* = 3). All procedures in this study were conducted after obtaining approval from the Institutional Review Board of Gangneung-Wonju National University (Approval No. GWNUIRB-2016-26-1).

### Procedures

Initially, the height (cm), weight (kg), and body mass index (BMI) of included elderly individuals were measured. The study participants were asked to keep their regular daily patterns and to wear a triaxial accelerometer for two weeks beginning with the day after their body measurements. A day after completing their physical activity measurements using the accelerometer, this study measured hand grip strength (strength), chair stand (muscular endurance), sit and reach (flexibility), timed up and go (balance), walking around two cones in a figure 8 (coordination), 2-min marching in place (cardiovascular and respiratory endurance), and 6-min walking (walking ability), which were aspects of elderly health included in the National Fitness Award 100 in Korea.

### Measurements

#### Physical frailty

"Gait speed" was reported to be the most important predictor of physical aging among the five measurement items21. As such, a 5-m walking test has been conducted to identify elderly who were physically healthy, if they had a record of 2.3 sec or less.

#### Body mass index (BMI)

The height and weight were measured using an analog extensometer and a regular digital weight scale; body mass index was calculated using the equation: weight (kg) / squared height (m2).

#### Daily physical activity

For physical activity measurement, a triaxial accelerometer (Active Style Pro HJA-350IT; Omron Health Care Co., Ltd., Kyoto, Japan) with verified reliability and validity was used^19, 22, 23^; the device was worn on the right side of the waist of the participants. The subjects were monitored over two weeks, including five weekdays and two days of the weekend. The days during which the accelerometer was worn for less than 600 min per day were excluded and the weekly measurement data set was considered valid if the device was worn for at least three weekdays and one day of the weekend^24, 25^. Metabolic equivalents (METs) were classified using the following criteria: 0.9 METs < sedentary behavior ≤ 1.5 METs, 1.5 METs < light physical activity < 3.0 METs, 3.0 METs ≤ moderate physical activity < 6.0 METs, vigorous physical activity ≥ 6.0 METs, moderate to vigorous physical activity ≥ 3.0 METs. The accelerometers used in this study were manufactured for average adults; previous studies have confirmed the accuracy of these devices if physical activity intensity and step measurement algorithm was used for physically healthy elderly individuals without walking issues^22, 23^.Moreover, the accelerometers used in this study allowed estimation of intensity by activity, dividing the types of activity into locomotive activity and non-locomotive activity^18, 19^.

#### Functional fitness

Functional fitness included the items for elderly physical activity test from National Fitness Award 100 in Korea, used for ensuring the health of the citizens in Korea^5^; the validity and reliability of these items were verified by Choi et al. (2014) ^26^. Therefore, this study has measured a total of six items, summarizing the results of the existing literature. An overview of measurement methods is presented as follows. The hand grip strength measurement involved pulling on a dynamometer as hard as possible for 5 sec. This test was repeated twice for left and right hands; the larger value of the two measurements was selected and entered with a precision of 0.1 kg. The relative grip strength was calculated using the following equation: hand grip strength (kg) / weight (kg) × 100. Chair stand, indicating muscular endurance, involved fully standing up and fully sitting down for 30 sec. The test was performed twice, and the larger value was selected. Sit and reach, which indicates flexibility, involved the lowering of the upper body and extending the two hands to push the measuring board as far as possible. The test was performed twice, and the larger value was selected in 0.1 cm increments. Timed up and go, which indicates balance, was measured using a cone placed so that the distance between a fixed chair to the backside of the cone was 3 m; when the participants were given the start signal, they were asked to stand up from the chair, walk as fast as possible to the cone and circle the cone to return to their chair. The test was performed twice, and the larger value was selected in 0.1 sec increments. Walking around two cones arranged to resemble the figure 8, which indicates coordination, involved marking the 1.6 m mark from the side of the chair, and placing cones at 1.8 m distances to the left and right of the cones, marking their furthest sides; after the start signal, the participants were asked to circle around the cone to their right side to sit down, and then stand immediately after to circle around the cone on their left hand side to approach the chair to sit down. The test was performed twice, and the larger value was selected in 0.1 sec increments. The 2-min marching in place, which indicates cardiovascular and respiratory endurance, involved the participants starting to march using their right foot with their knees reaching beyond a certain height. The test was performed once, and one measurement was recorded when the participant engaged in a proper walk with both feet. The 6-min walking test, showing walking ability, involved the participants walking as far as possible over 6 min. They were asked to stop when 6 min had past; the distance they had walked was measured in 0.1 m increments.

### Statistics

SPSS ver. 21.0 (SPSS Inc., Chicago, IL, USA) was used for all statistical analyses performed to verify the hypothesis set in this study. The mean and the standard deviations of the variables were calculated by descriptive statistical analysis; to comparatively analyze elderly fitness according to their fitness level, independent t-tests were performed. Pearson’s correlation analysis was conducted to identify the relationship between the level of physical activity and elderly fitness. The significance level of hypothesis verification was set at *p* < 0.05.

## RESULTS

The mean age, height, weight, and BMI of the study participants were 71.5 ± 3.3 years, 164.9 ± 4.6 cm, 65.2 ± 7.0 kg, and 24.1 ± 2.1 kg/m2, respectively.

The physical activity characteristics are shown in Table 1. The mean number of steps performed by a healthy elderly man was 7567.5 ± 3316.8 steps per day. The length of locomotive physical activity per day averaged 135.8 ± 43.1 min; the other parameters were as follows: sedentary 0.0 ± 0.0 min, low intensity 92.3 ± 33.7 min, moderate intensity 43.0 ± 26.1 min, vigorous intensity 0.4 ± 1.6 min, and MVPA 43.4 ± 26.3 min. The length of non-locomotive physical activity per day averaged 672.5 ± 69.1 min; the other parameters were as follows: sedentary 388.9 ± 81.3 min, low intensity 261.7 ± 58.7 min, moderate intensity 22.4 ± 13.4 min, and MVPA 22.4 ± 13.4 min. The length of total physical activity per day averaged 808.3 ± 69.5 min; specifically, sedentary was 388.9 ± 81.3 min, low intensity 354.1 ± 71.7 min, moderate intensity 65.4 ± 29.7 min, vigorous intensity 0.4 ± 1.6 min, and MVPA 65.9 ± 29.8 min.

**Table 1.** Physical activity characteristics of participants.

Physical activity	Total (n = 22)
Steps (counts/day)	7567.5 ± 3316.8
**Locomotion**	
Sedentary activity (min/day)	0.0 ± 0.0
Light activity (min/day)	92.3 ± 33.7
Moderate activity (min/day)	43.0 ± 26.1
Vigorous activity (min/day)	0.4 ± 1.6
MVPA (min/day)	43.4 ± 26.3
Total activity (min/day)	135.8 ± 43.1
**Non-locomotion**	
Sedentary activity (min/day)	388.9 ± 81.3
Light activity (min/day)	261.7 ± 58.7
Moderate activity (min/day)	22.4 ± 13.4
Vigorous activity (min/day)	0.0 ± 0.0
MVPA (min/day)	22.4 ± 13.4
Total activity (min/day)	672.5 ± 69.1
**Total (Locomotion + Non-locomotion)**	
Sedentary activity (min/day)	388.9 ± 81.3
Light activity (min/day)	354.1 ± 71.7
Moderate activity (min/day)	65.4 ± 29.7
Vigorous activity (min/day)	0.4 ± 1.6
MVPA (min/day)	65.9 ± 29.8
Total activity (min/day)	808.3 ± 69.5

All values are presented as means ± standard deviation

MVPA: Moderate to vigorous physical activity, METs: metabolic equivalents

0.9 METs < sedentary behavior ≤ 1.5 METs, 1.5 METs < light physical activity < 3.0 METs, 3.0 METs ≤ moderate physical activity < 6.0 METs, vigorous physical activity ≥ 6.0 METs, moderate to vigorous physical activity ≥ 3.0 METs, Total activity corresponds to > 0.9 METs.

The results of analysis of physical activity characteristics of the study participants are shown in Table 2. Evaluating flexibility, sit and reach (9.9 ± 9.2 cm), and coordination, timed up and go (5.7 ± 0.7 sec), were measured at Grade 1, which is the highest level for National Fitness Award 100 in Korea standard data. Next, comparative grip strength (52.0 ± 7.8%) which evaluates strength, chair stand (20.7 ± 4.2 reps), which evaluates muscular endurance, walking around two cones in a figure 8 (22.1 ± 3.3 sec) which evaluates coordination and 6-min walking (618.7 ± 54.3 m), which evaluates walking ability, were located between Grade 1 and 2 of the National Fitness Award 100 standard data. On the other hand, 2-min marching in place (87.6 ± 14.6 reps), which evaluates cardiovascular and respiratory endurance, was Grade 3 from the National Fitness Award 100 standard data.

**Table 2.** Physical fitness characteristics of participants

Physical fitness	Total (n = 22)	Fitness standards (average)		
		1^st^ Grade	2^nd^ Grade	3^rd^ Grade
Hand grip strength (%)	52.0 ± 7.8	53.6	48.3	43.1
Chair stand (rep/30 sec)	20.7 ± 4.2	22.0	19.0	15.0
Sit and reach (cm)	9.9 ± 9.2	9.2	4.4	-0.4
Timed up and go (sec)	5.7 ± 0.7	5.6	6.4	7.2
Walking around two corns in a figure 8 (sec)	22.0 ± 3.3	22.5	25.6	28.7
2-min Marching in place (rep)	87.6 ± 14.5	116.0	103.0	90.0
6-min Walking (m)	618.7 ± 54.3	644.0	599.0	555.0

All values are presented as means ± standard deviation

Fitness standards were used to categorize functional fitness levels into 3 grades: 1^st^ grade is the highest and 3^rd^ grade is the lowest. Fitness standards were based on the 2015 Korea National Fitness Award for Elderly.

Based on the "High" level criterion for division proposed by the Korean Golden Age Forum (2010) of 6,500 steps (counts/day) [27], Table 3 shows the division of participants in inactive (*n* = 9) and active elderly men (*n*=13). The results indicated significantly higher results in 2-min marching in place, related to cardiovascular and respiratory endurance, for active elderly men compared to their inactive counterparts (*p* < 0.05). However, no significant differences were found between the two groups on other fitness items. Based on a 60-min MVPA per day proposed by the Japanese Ministry of Health, Labor and Welfare for better measurement of elderly health as the criteria for division, Table 4 shows the division of study participant in inactive (*n* = 11) and active elderly men (*n* = 11). The results indicated significantly higher results in 2-min marching in place for active elderly men, related to cardiovascular and respiratory endurance, compared to their inactive counterparts (*p* < 0.05). However, no significant differences were detected between the two groups in other fitness items.

**Table 3.** Comparison of functional fitness between physically active and inactive participants.

PA (steps/day)	Total (n = 22)	Physically inactive (n = 9)	Physically active (n = 13)	t	p value
Fitness					
Hand grip Strength (%)	52.0 ± 7.8	51.4 ± 10.1	52.4 ± 6.3	-0.29	0.77
Chair stand (rep/30 sec)	20.7 ± 4.2	19.6 ± 2.9	21.5 ± 4.9	-1.08	0.29
Sit & reach (cm)	9.9 ± 9.2	11.7 ± 9.6	8.5 ± 9.0	0.79	0.44
Timed up & go (sec)	5.7 ± 0.7	5.9 ± 0.6	5.6 ± 0.8	1.06	0.30
Walking in a figure 8 (sec)	22.0 ± 3.3	23.2 ± 4.4	21.2 ± 2.1	1.39	0.18
2-min Marching in place (rep)	87.6 ± 14.5	78.9 ± 13.3	93.6 ± 12.5	-2.65	0.02*
6-min Walking (m)	618.7 ± 54.3	602.0 ± 71.0	630.4 ± 37.9	-1.22	0.24

All values are presented as means ± standard deviation

MVPA: Moderate to vigorous physical activity

Physical inactive corresponds to < 60-min of daily MVPA, physical active corresponds to ≥ 60-min of daily MVPA.

*p < 0.05

**Table 4.** Comparison of functional fitness between physically active and inactive participants.

PA (steps/day)	Total (n = 22)	Physically inactive (n = 11)	Physically active (n = 11)	t	p value
Fitness					
Hand grip Strength (%)	52.0 ± 7.8	52.6 ± 9.5	51.4 ± 6.1	0.34	0.74
Chair stand (rep/30 sec)	20.7 ± 4.2	19.6 ± 2.5	22.5 ± 4.9	-2.06	0.05
Sit & reach (cm)	9.9 ± 9.2	9.8 ± 10.9	9.9 ± 7.6	-0.03	0.97
Timed up & go (sec)	5.7 ± 0.7	5.6 ± 0.8	5.8 ± 0.7	-0.57	0.57
Walking in a figure 8 (sec)	22.0 ± 3.3	22.1 ± 4.4	22.0 ± 1.9	0.03	0.98
2-min Marching in place (rep)	87.6 ± 14.5	81.4 ± 13.7	93.8 ± 13.1	-2.18	0.04*
6-min Walking (m)	618.7 ± 54.3	607.4 ± 66.7	630.1 ± 38.2	-0.98	0.34

All values are presented as means ± standard deviation

MVPA: Moderate to vigorous physical activity

Physical inactive corresponds to < 60-min of daily MVPA, physical active corresponds to ≥ 60-min of daily MVPA.

*p < 0.05

Table 5 shows the comparative analysis between the daily life physical activity and elderly fitness of healthy elderly men. Regarding the relationship between daily physical activity and elderly fitness, the 2-min marching in place, which evaluates walking ability and cardiovascular and respiratory endurance, was significantly positively correlated with elderly fitness, r = 0.63 (*p* < 0.01); total MVPA and 2-min marching in place were also found positively correlated, r = 0.46 (p < 0.05), r= 0.47 (*p* < 0.05). Moreover, a positive correlation was also found between locomotive MVPA and 2-min marching in place, *r* = 0.50 (*p*<0.05); however, no relationship with other fitness items was detected. Particularly, non-locomotive physical activity had no relationship with fitness items.

**Table 5.** Associations of daily physical activity with functional fitness in elderly men.

		Hand grip strength	Chair stand	Sit & reach	Timed up amp; go	Walking in a figure 8	2-min Marching in place	6-min Walking
Steps	r	0.07	0.36	-0.03	-0.06	-0.24	0.63 **	0.22
Locomotion Sedentary behavior	r	-	-	-	-	-	-	-
Locomotion Light PA	r	0.29	0.07	0.05	0.08	-0.03	0.09	0.03
Locomotion Moderate PA	r	-0.10	0.30	-0.07	-0.02	-0.24	0.49 *	0.14
Locomotion Vigorous PA	r	0.21	0.05	-0.18	-0.15	-0.09	0.27	0.14
Locomotion MVPA	r	-0.08	0.30	-0.08	-0.03	-0.24	0.50 *	0.14
Locomotion total PA	r	0.24	-0.02	0.04	-0.17	0.38	0.11
Non-loco Sedentary behavior	r	-0.08	0.06	0.07	-0.07	0.21	-0.17	-0.11
Non-loco Light PA	r	0.08	0.01	0.23	0.09	-0.34	0.31	0.22
Non-loco Moderate PA	r	0.03	0.05	0.26	0.13	-0.21	0.07	0.24
Non-loco Vigorous PA	r	-	-	-	-	-	-	-
Non-loco MVPA	r	0.03	0.05	0.26	0.13	-0.21	0.07	0.24
Non-loco total PA	r	-0.01	0.10	0.33	0.00	-0.08	0.09	0.12
Total Sedentary behavior	r	-0.08	0.06	0.07	-0.07	0.21	-0.17	-0.11
Total Light PA	r	0.20	0.04	0.21	0.11	-0.30	0.29	0.20
Total Moderate PA	r	-0.07	0.29	0.05	0.04	-0.30	0.46 *	0.23
Total Vigorous PA	r	0.21	0.05	-0.18	-0.15	-0.09	0.27	0.14
Total MVPA	r	-0.06	0.29	0.04	0.03	-0.31	0.47 *	0.23
Total PA	r	0.10	0.25	0.32	0.03	-0.19	0.32	0.19

PA: Physical activity, MVPA: Moderate to vigorous physical activity, Non-loco: Non-locomotive, Total: Locomotive activity + Non-locomotive activity 0.9 METs < sedentary behavior ≤ 1.5 METs, 1.5 METs < light physical activity < 3.0 METs, 3.0 METs ≤ moderate physical activity < 6.0 METs, vigorous physical activity ≥ 6.0 METs, moderate to vigorous physical activity ≥ 3.0 METs, Total activity corresponds to>0.9 METs.

*p < 0.05

**p < 0.01

## DISCUSSION

This study has evaluated healthy elderly men using a triaxial accelerometer to measure a variety of physical activity variables and analyze their relationships with elderly fitness. The results indicated that active elderly individuals with high step count and MVPA, and specifically locomotive MVPA, showed high performance in 2-min marching in place, which evaluated cardiovascular and respiratory endurance. However, non-locomotive physical activities that mainly involved the upper body did not show any correlation with items such as grip strength.

Elderly individuals have a high risk of a variety of diseases due to decreasing functions and strength with age; these conditions eventually pile up until they die^28, 29^. It has been reported that improving fitness through increasing physical activity volume can decrease a variety of risk factors relating to health^30^. The average number of steps for elderly individuals aged 71 was 7,568, which was higher than 6,500~7,000 steps for those in their 60s presented by the Korean Golden Age Forum. The number of steps in our study were almost equivalent to that mentioned in the guidelines of the Japanese Ministry of Health, Labor and Welfare of 7,000 steps per day for those in their 70s. Moreover, the average time spent on MVPA per day was 65 min; this result was higher than the recommendations by Health Japan 21, which recommended 60 min of MVPA per day for those aged between 18 to 64^31^. While no clear guidelines for daily recommended MVPA are currently available, elderly individuals from United Kingdom aged 65 years or above are recommended to engage in MVPA for 30 min a day, at least five times a week; in the case of cardiovascular diseases, the recommended duration of MVPA is at least 150 min, with the focus on cardio physical activities of over 10 min in duration ^32^. The average duration of MVPA in healthy Japanese elderly individuals with an average age of 74.4 years was 50 min per day^14^. A study using a typical sample of Portuguese elderly individuals with an average age of 74.2 years reported that the duration of their MVPA averaged 31.5 min per day^16^. For elderly individuals in the United Kingdom and Italy with an average age of 76.1 years, a study reported that the duration of their MVPA averaged 23.8 min per day^33^. Therefore, the participants of this study tended to be more intensely active from the perspective of daily physical activity.

In our previous study we analyzed daily physical activity related variables in boys aged 9 to 12 years and detected that the locomotive physical activity duration for boys averaged 203 min per day^34^. On the other hand, the locomotive hours for the elderly men covered in this study averaged 163 min, which was about 40 min less than the 203 min spent by boys on locomotive physical activity. These results indicate that aging leads to lower locomotive physical activity, which leads to the need for efforts and further studies on increasing the locomotive physical activity, as well as on effectively improving the non-locomotive physical activity duration such as household chores.

It has been previously reported that increasing cardiovascular and respiratory endurance in elderly individuals can reduce the risk of cardiovascular disease; additionally, improving strength and muscular endurance and flexibility leads to improvements in daily life abilities, such as posture stability and functional skills ^35^. As such, the Ministry of Culture, Sports and Tourism and the Korea Institute of Sports Science have been conducting Surveys on National Physical Fitness (2015) to identify fitness factors and levels by age^5, 36^. Compared to the fitness qualification standards proposed by the National Fitness Award 100 in 2015, the 2-min marching in place that evaluated cardiovascular and respiratory endurance was at the lowest grade, despite the fact that the participants were active in terms of MVPA. This study indicated that the average reps of 2-min marching in place for elderly men was 88 reps; the number of reps of 2-min marching in place identified by Park et al. (2016) for healthy elderly women using an aging measurement tool was 106 reps, resulting in a number of reps lower than that previously recorded for elderly women. This appears to be due to the fact that Park et al. (2016) included individuals who regularly engaged in physical activity as it targeted all women in Korea^9^; however, this study has excluded individuals who engaged in strenuous exercise and high-intensity physical activity and limited its participants to elderly men residing in urbanized areas. As continuous moderate cardio physical activities over 10 min remain the core of cardiovascular and respiratory endurance^37, 38^, the participants of this study might not have included continuous moderate cardio physical activities in their daily MVPA of over 60 min. In other words, it appears that the 60 min of MVPA for the participants of this study consisted of accumulations of short MVPA increments of 1-2 min. However, this study indicated a moderate-level significant positive correlation between 2-min marching in place, evaluating cardiovascular and respiratory endurance, and the number of steps and duration of MVPA and locomotive MVPA in this study. Therefore, moderate to vigorous cardio physical activities > 10 min were found effective for improving cardiovascular and respiratory endurance in elderly individuals; however, elderly individuals who are unable to engage in exercises due to reasons, such as lower fitness and surrounding environment, it appears that raising moderate or more intense physical activity such as walking continuously would be somewhat helpful in improving cardiovascular and respiratory endurance or reducing the risk of it becoming lower. It was previously reported that the largest portion of daily MVPA for elderly individuals was walking, followed by household chores^39^. It has also been reported that elderly individuals have significantly lower continuous MVPA lasting for over 10 min compared to younger individuals. Therefore, future research efforts should also focus on comparing individuals with more daily MVPA that continues for over 10 min, with individuals with short bursts of MVPA lasting for 1-2 min with more study participants, studying their relationships with cardiovascular and respiratory endurance.

Corcoran et al. (2016) studied elderly individuals residing in long-term care homes, dividing them into four groups of active and comparatively inactive individuals, and reported that inactive individuals had lower grip strength and cardiovascular and respiratory endurance. Comparing the results from Corcoran et al. (2016) and the results of this study, the results were similar for cardiovascular and respiratory endurance, but different for grip strength, as this study did not find a correlation with variables relating to physical activity. The accelerometer used in this study had a special algorithm that allowed for the upper body-focused physical activities evaluation, such as cleaning, (mopping and vacuuming), doing the laundry, and washing the sheets^19^; therefore, the expectation was that the moderate or vigorous upper-body related physical activities would be related to grip strength. However, several reasons might have led to the absence of this correlation. The grip strength of the study participants was in the highest grades of the standards of Korean elderly individuals. Corcoran et al. (2016) studied elderly individuals with 25 min of MVPA per week and 2,150 steps per day; therefore, it can be expected that their participants would have had lower locomotive physical activity and comparatively more non-locomotive, or upper body-focused, physical activity. Moreover, the upper body-related physical activity such as household chores for elderly individuals such as those included by Corcoran et al. (2016) would heavily influence grip strength. Therefore, it would be important to include more participants who are much more weakened from aging, as well as those who are healthy to study the locomotive and non-locomotive physical activity and their relationship with elderly health.

This study had limitations. The participants of this study were limited to elderly men from a single city; moreover, the small number of participants has made it difficult to generalize obtained results to all elderly men across the country. However, this study was the first of its kind to classify locomotive and non-locomotive physical activity in order to understand the relationship of physical activity with fitness in the elderly; moreover, the use of an accelerometer with a special algorithm to measure the variables of physical activity is a key strength of this study.

Summarizing the results of this study, the daily physical activity, and specifically the number of steps and locomotive MVPA were related to cardiovascular and respiratory endurance. However, no correlations were detected among upper body-related, non-locomotive physical activity, and elderly fitness. Going forward, it would be important to expand the scope of this study to include participants of all ages of elderly individuals and of all regions; moreover, it is necessary to conduct a future study on the efficacy of accelerometers worn on the wrist, as well as those used in this study for elderly individuals who face higher levels of upper body-focused, or sedentary physical activity.
